# WGS based analysis of acquired antimicrobial resistance in human and non-human *Acinetobacter baumannii* isolates from a German perspective

**DOI:** 10.1186/s12866-021-02270-7

**Published:** 2021-07-10

**Authors:** Gamal Wareth, Christian Brandt, Lisa D. Sprague, Heinrich Neubauer, Mathias W. Pletz

**Affiliations:** 1 Friedrich-Loeffler-Institut, Institute of Bacterial Infections and Zoonoses, Naumburger Str. 96a, 07743 Jena, Germany; 2 Institute for Infectious Diseases and Infection Control, Jena University Hospital, Am Klinikum 1, 07747 Jena, Germany; 3 Department of Bacteriology, Faculty of Veterinary Medicine, Benha University, Moshtohor, Toukh, 13736 Egypt; 4 Research Campus Infectognostics, Philosophenweg 7, 07743 Jena, Germany

**Keywords:** A. baumannii, Acquired resistance, WGS, NCBI, Germany

## Abstract

**Background:**

*Acinetobacter baumannii* ability to develop and acquire resistance makes it one of the most critical nosocomial pathogens globally. Whole-genome sequencing (WGS) was applied to identify the acquired or mutational variants of antimicrobial resistance (AMR) genes in 85 German *A. baumannii* strains utilizing Illumina technology. Additionally, the whole genome of 104 German isolates deposited in the NCBI database was investigated.

**Results:**

*In-silico* analysis of WGS data revealed wide varieties of acquired AMR genes mediating resistance mostly to aminoglycosides, cephalosporins, carbapenems, sulfonamides, tetracyclines and macrolides. In the 189 analyzed genomes, the *ant* (3″)-IIa conferring resistance to aminoglycosides was the most frequent (55%), followed by *bla*_ADC.25_ (38.6%) conferring resistance to cephalosporin, *bla*_OXA-23_ (29%) and the *bla*_OXA-66_ variant of the intrinsic *bla*_OXA-51-likes_ (26.5%) conferring resistance to carbapenems, the *sul*2 (26%) conferring resistance to sulfonamides, the *tet.* B (19.5%) conferring resistance to tetracycline, and *mph*. E and *msr.* E (19%) conferring resistance to macrolides. *bla*_TEM_ variants conferring resistance to cephalosporins were found in 12% of genomes. Thirteen variants of the intrinsic *bla*_OXA-51_ carbapenemase gene, *bla*_OXA-510_ and *bla*_ADC-25_ genes were found in isolates obtained from dried milk samples.

**Conclusion:**

The presence of strains harboring acquired AMR genes in dried milk raises safety concerns and highlights the need for changes in producing dried milk. Acquired resistance genes and chromosomal gene mutation are successful routes for disseminating AMR determinants among *A. baumannii.* Identification of chromosomal and plasmid-encoded AMR in the genome of *A. baumannii* may help understand the mechanism behind the genetic mobilization and spread of AMR genes.

**Supplementary Information:**

The online version contains supplementary material available at 10.1186/s12866-021-02270-7.

## Background

*Acinetobacter baumannii* (*A. baumannii*) is a member of the ESKAPE pathogens, the leading cause of multidrug-resistant (MDR) and extensively drug-resistant (XDR) nosocomial infections worldwide [1]. The emergence of MDR *A. baumannii* strains resistant to last-resort antibiotics such as carbapenems and colistin is on the rise in hospital settings globally and complicates the treatment [2]. Therefore, the World Health Organization (WHO) has classified *A. baumannii* among the most dangerous MDR pathogens worldwide. It is considered one of the critical pathogens that need developing new antibiotics [3, 4]. In Germany, *A. baumannii* is a ubiquitous pathogen, and several communities and hospital-based outbreaks were reported in 13 out of 16 federal states [5]. Among other sources, the pathogen was also isolated from companion animals [6] and found in dried milk samples [7]. Besides, *A. baumannii* was released via manure [8] and through wastewater treatment plant (WWTP) effluents [9] into the environment in various districts of Germany. Still, the current knowledge on antibiotic resistance in strains collected from non-humans origin is scarce [10].

*Acinetobacter baumannii* possesses the ability to develop intrinsic resistance via reducing membrane permeability, efflux pump activity, and the production of wide varieties of ß-lactamases enzymes [11]. However, resistance in this pathogen is frequently associated with mobile genetic elements (MGEs) transferable between bacteria, enabling rapid dissemination and maintenance of resistance genes between different bacterial species [12]. It can also acquire resistance via mutational changes in chromosomal structure and horizontal gene transfer [13], in addition to some different naturally occurring intrinsic resistance genes [14]. *Acinetobacter baumannii* has an unprecedented ability to acquire resistance against antimicrobial agents from diverse sources and further disseminate and develop new resistance mechanisms [15]. Besides the massive resistance island coding for multiple intrinsic resistance within its genome, it can rapidly acquire further extrinsic resistance during antibiotic therapy by acquiring additional genetic determinants by cross-species horizontal gene transfer [16, 17]. The genome of *A. baumannii* consists of a chromosome and various plasmids. Most of them have been linked to the acquisition of AMR genes [18]. Comparative genomic analysis of *A. baumannii* strains revealed that the genome of *A. baumannii* could acquire a large amount of foreign DNA, which could play a role in antimicrobial resistance and pathogenesis [19, 20]. Thus, the current study is dedicated to collect data on acquired resistance genes in 85 clinical and non-clinical *A. baumannii* strains originating from Germany. Moreover, the resistance profile in another 104 genomes of German *A. baumannii* strains deposited in the National Centre for Biotechnology Information (NCBI) database was investigated.

## Results

### The phenotyping characterization of *A. baumannii*

**Fig. 1:**
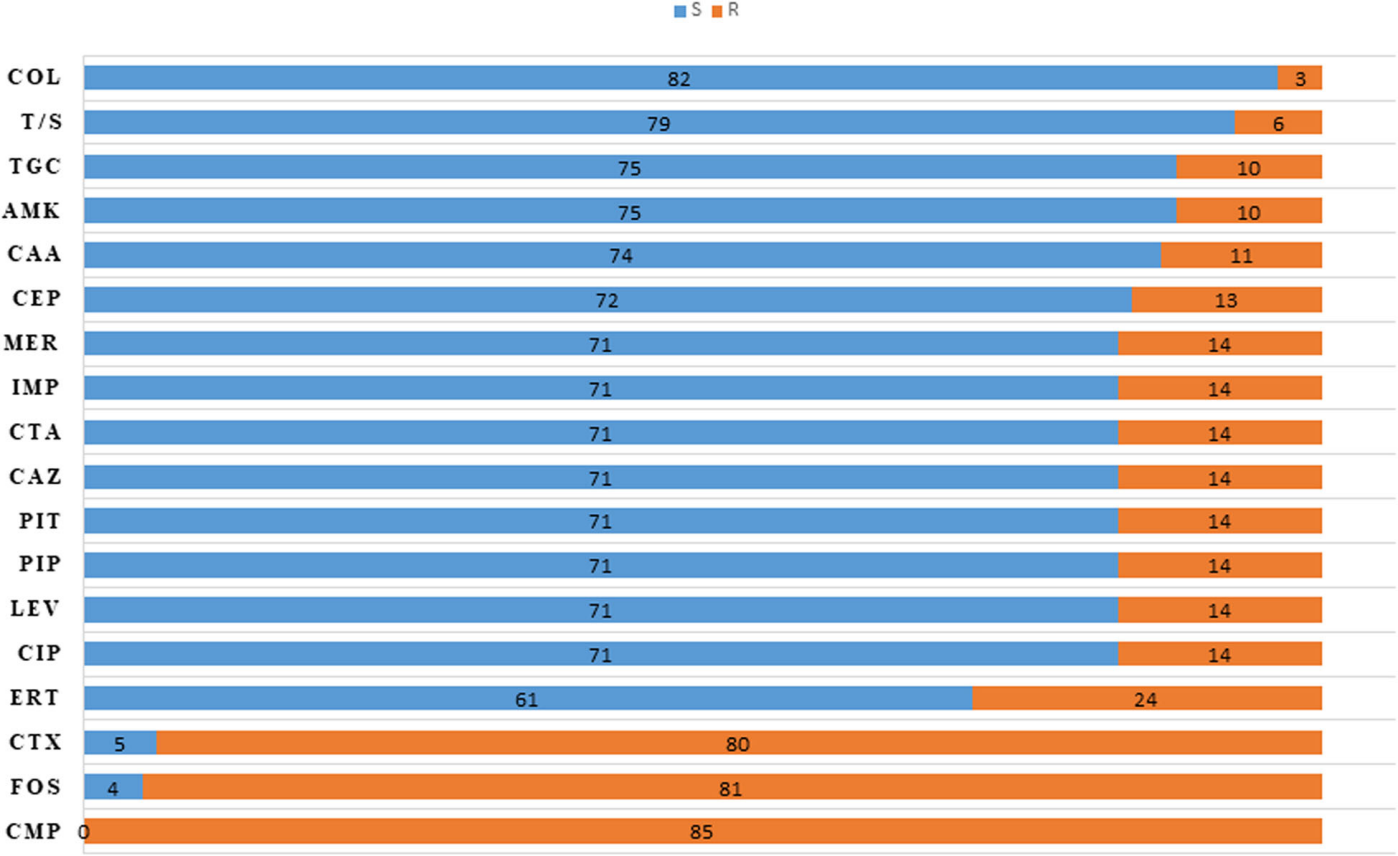
Number of resistant and sensitive isolates among 85 *A. baumannii* strains isolated from human and milk powder samples in Germany. COL, Colistin; T/S, Trimethoprim/Sulfamethoxazole; TGC, Tigecycline; AMK, Amikacin; CAA, Ceftazidime/Avibactam; CEP, Cefepime; MER, Meropenem; IMP, Imipenem; CTA, Ceftolozane/Tazobactam; CAZ, Ceftazidime; PIT, Piperacillin/Tazobactam; PIP, Piperacillin; LEV, Levofloxacin; CIP, Ciprofloxacin; ERT, Ertapenem; CTX, Cefotaxime; FOS, Fosfomycin; CMP, Chloramphenicol

**Fig. 2:**
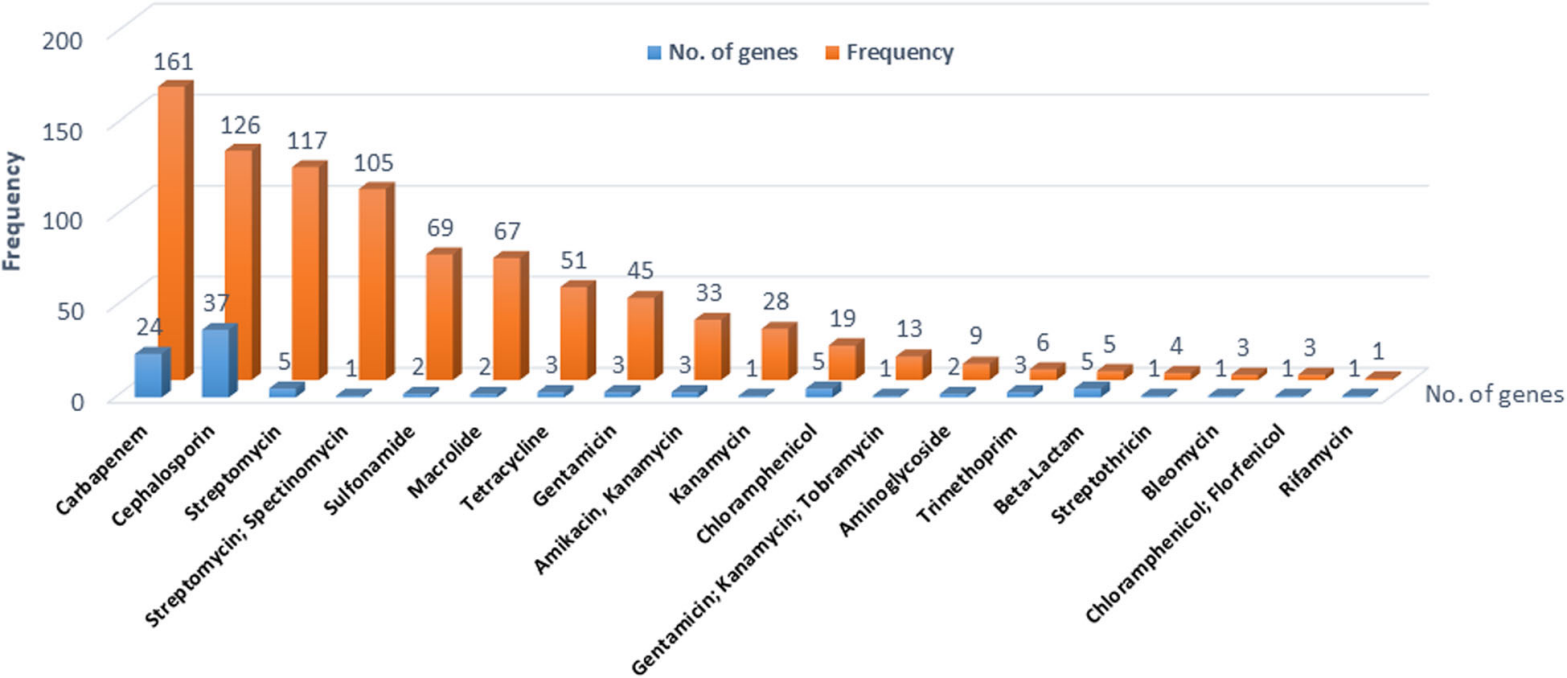
Number and frequency of AMR genes harbored within 104 genomes of German *A. baumannii* isolates obtained from the NCBI database as of September 2020

The phenotyping characterization of 85 *A. baumannii* isolates showed a high frequency of resistance for chloramphenicol (100%), followed by fosfomycin in 81 (95%) isolates and the third-generation cephalosporins, cefotaxime in 80 (94%) isolates. Resistance to at least one of the carbapenem compounds was found in 24 (28%) isolates. Resistance to aminoglycosides (amikacin) and tetracycline (tigecycline) was found in 10 (11%) isolates to each. The lowest frequency of resistance was seen for colistin in three isolates (Fig. 1). In parallel, the analysis of the downloaded 104 whole-genome of *A. baumannii* deposited at the NCBI indicates that the strains harbored genes mediating resistance to ten antimicrobial agent groups, including ß-lactams, (carbapenems and cephalosporins), aminoglycosides, phenicoles, tetracycline, trimethoprim, sulfonamides, macrolides, streptothricin, bleomycin and rifampicin. The frequency of resistance toward aminoglycosides was the highest, followed by carbapenems and cephalosporins. The lowest frequency was seen for streptothricin, bleomycin and rifampicin (Fig. 2).

### *In-silico* detection of acquired AMR genes in *A. baumannii* strains

The *in-silico* detection of acquired AMR genes in *A. baumannii* isolates (*n* = 85) based on WGS data using the ResFinder database succeeded in identifying 40 acquired AMR genes (Supplementary Table 1). Twenty-two different β-lactamases resistance genes belonging to three different Ambler classes were identified. Thirteen genes were identified in isolates obtained from dried milk samples. Seven genes were identified in clinical isolates obtained from humans, and two genes were shared in isolates obtained from milk samples and humans. At least one, two, and 19 different variants of class C, A, and D β- lactamases were identified, respectively. The Ambler class D β-lactamases were the most predominant genes and represented in 19 *bla*_OXA_ ß-lactamases variants. Among them, 16 gene variants were belonging to the intrinsic *bla*_OXA-51-like_ carbapenemase group, of which the *bla*_OXA.430_ gene was most frequent, present in 24 (28.2%) isolates obtained from milk powder. All strains harbored *bla*_OXA.430_ gene were resistant to cefotaxime, and four of them showed resistance to ertapenem. This was followed by *bla*_OXA.91_ and *bla*_OXA.66_ genes of the same *bla*_OXA-51-like_ group and were found in 21 (24.5%) and ten (11.8%) isolates, respectively. Four isolates (4.7%) obtained from milk powder samples harbored *bla*_OXA.343_ gene of *bla*_OXA-51-like_; however, they showed sensitivity to all tested antibiotics. Additionally, the *bla*_OXA.23_ was found in 12 (14%) isolates of human origin, and a single variant of *bla*_OXA-510_ and *bla*_OXA-521_, each was found in one isolate (Table 1).

**Table 1:** List of acquired β-lactamases resistance genes identified in *A. baumannii* isolates (*n* = 85) from humans and dried milk based on WGS data using ResFinder database

Mechanism	AMR genes	Group	No. (%)	Source	Resistance pattern
Amber class A β-lactamases	*bla*TEM.1D_1	TEM	3 (3.5%)	Human	PIP-PIT/CTX-CAZ-CAA-CTA-CEP /IMP-MER-ERT
	*bla*CARB.5_1	CARB-5	3 (3.5%)	Human	
Amber class C β-lactamases	*bla*ADC.25_1	ADC	14 (16.5%)	Human	11 [PIP-PIT/CTX-CAZ-CTA-CEP/IMP-MER-ERT]
			71 (83.5%)	Milk powder	66 [CTX], 10 [ERT]
Amber class D β-lactamases	*bla*OXA.23_1	OXA-23	12 (14%)	Human	12 [PIP-PIT/CTX-CAZ-CTA-CE/IMP-MER-ERT], 10 [CAA]
	blaOXA.120_1	OXA-51	2 (2.3%)	Milk powder	CTX
	*bla*OXA.203_1		1 (1.2%)	Milk powder	CTX
	*bla*OXA.259_1		1 (1.2%)	Milk powder	CTX-ERT
	*bla*OXA.343_1		4 (4.7%)	Milk powder	–
	*bla*OXA.346_1		4 (4.7%)	Milk powder	4 [CTX], 2 [ERT]
	*bla*OXA.380_1		2 (2.3%)	Milk powder	CTX
	*bla*OXA.386_1		1 (1.2%)	Milk powder	CTX
	*bla*OXA.424_1		1 (1.2%)	Milk powder	CTX-ERT
	*bla*OXA.430_1		24 (28.2%)	Milk powder	24 [CTX], 4 [ERT]
	*bla*OXA.431_1		1 (1.2%)	Milk powder	CTX
	*bla*OXA.51_1		1 (1.2%)	Milk powder	CTX
	*bla*OXA.64_1		9 (10.5%)	Milk powder	8[CTX]/1 [COL]
	*bla*OXA.66_1		10 (11.8%)	Human	10 [PIP-PIT/CTX-CAZ-CTA/IMP-MER-ERT], 9 [CEP], 7 [CAA].
	*bla*OXA.69_1		1 (1.2%)	Human	PIP-PIT/CTX-CAZ-CAA-CTA-CEP /IMP-MER-ERT
	*bla*OXA.72_1		1 (1.2%)	Human	PIP-PIT/CTX-CAZ-CTA/IMP-MER-ERT
	*bla*OXA.91_1		2 (2.2%)	Human	PIP-PIT/CTX-CAZ-CAA-CTA-CEP /IMP-MER-ERT
			19 (22.3%)	Milk powder	CTX
	*bla*OXA.510_1	single variant	1 (1.2%)	Milk powder	CTX
	*bla*OXA.521_1	single variant	1 (1.2%)	Human	PIP-PIT/CTX-CAZ-CAA-CTA-CEP/IMP-MER-ERT

*AMR* antimicrobial resistance gene, *PIP* piperacillin, PIT piperacillin/tazobactam, CTX cefotaxime, CAZ ceftazidime, CAA ceftazidime/avibactam, CEP cefepime; CTA ceftolozane/tazobactam, IMP imipenem, MER meropenem, ERT ertapenem

The Ambler class C β-lactamases, *Acinetobacter*-derived cephalosporinase *bla*_ADC.25_, was identified in all isolates (100%). Among them, 11 isolates (13%) were carbapenem-resistant, and 80 (94%) isolates were resistant to the third-generation cephalosporin cefotaxime antibiotic. Two acquired AMR genes belonging to the Ambler class A β-lactamases were identified. The *bla*_TEM.1D_ was found in three isolates (3.8%), and carbenicillin hydrolyzing β-lactamase *bla*_CARB.5_ was found in another three isolates. All six isolates were resistant to cephalosporins and carbapenems (Table 1).

On the other hand, 18 non-β-lactamases AMR genes conferring resistance to aminoglycosides, tetracyclines, phenicoles, sulfonamides and macrolides were identified. None of them was found in isolates obtained from milk samples. Eight aminoglycoside-modifying enzymes (AMEs) genes were detected. Among them, three were aminoglycoside acetyltransferase (ACT), which were encoded by plasmids, transposons, and integron in *A. baumannii,* two were aminoglycoside nucleotidyltransferase (NUT), two were aminoglycoside phosphotransferase (PHT), and one was aminoglycoside methyltransferase (MET). Those eight AMEs genes conferred resistance to amikacin in ten isolates. At least three genes encoding resistance to each tetracycline and phenicoles compounds were identified. The *tet*. B encoding resistance to tetracycline was identified in nine isolates; among them, eight were resistant to tigecycline. In spite, *tet*.39 was identified in two isolates, but both were susceptible to tetracycline compounds. All investigated isolates were resistant to chloramphenicol; however, only four isolates harbored three genes (*cat*A, *cat*B and *flo*R) confer resistance to phenicoles were identified. Two genes encoding resistance to each macrolide and sulfonamide antibiotics were identified. The gene *sul*1 and *sul*2 variants were found in three and one isolates, respectively, and all were resistant to trimethoprim/sulphamethoxazole. The *mph*. E and *msr*. E genes encoding resistance to macrolides were identified in two and three isolates, respectively; however, none of them showed resistance to macrolides (Table 2).

**Table 2:** List of acquired non-β-lactamases resistance genes identified in *A. baumannii* isolates from humans (*n* = 14/85) based on WGS data using ResFinder databases

Antibiotic class	AMR resistant genes		Mechanism	Resistance pattern
	Gene family	Number (%)		
Aminoglycosides Antibiotic inactivation	*aac*.3...Ia_1	3 (21.5%)	ACT: Acetyltransferase	2/3 AMK
	*aac*.6...Iaf_1	1 (7%)	ACT: Acetyltransferase	AMK
	*aac*.6...Ian_1	1 (7%)	ACT: Acetyltransferase	AMK
	*ant.*2 … Ia_1	1 (7%)	NUT: Nucleotidyltransferase	AMK
	*aph*.3...Ia_7	6 (43%)	PHT: Phosphotransferase	AMK
	*aph*.6...Id_1	9 (64%)	PHT: Phosphotransferase	7/9 AMK
	*arm*A_1	7 (50%)	MET: Methyltransferase	AMK
	*str*A_1	9 (64%)	NUT: Nucleotidyltransferase	7/9 AMK
Phenicoles	*cat*A1_1	1 (7%)	Enzymes Inactivation	CMP
	*cat*B8_1	2 (14%)	Enzymes Inactivation	CMP
	*flo*R_2	1 (7%)	Antibiotic Efflux	CMP
Macrolide-lincosamide-streptogramin B (MLS)	*mph*. E_1	2 (14%)	Enzymes Inactivation	–
	*msr*. E_4	3 (21.4%)	Antibiotic Efflux	–
Sulfonamides	*sul*1_5	3 (21.4%)	Antibiotic Target Replacement	T/S
	*sul*2_2	1 (7%)	Antibiotic Target Replacement	T/S
Tetracyclines	*tet*.39._1	2 (14%)	Antibiotic Efflux	–
	*tet*. A_6	1 (7%)	Antibiotic Efflux	TGC
	*tet*. B_1	9 (64%)	Antibiotic Efflux	8/9 TGC

*AMK* amikacin, *CMP* chloramphenicol, T/S trimethoprim/sulfamethoxazole, TGC tigecycline

### *In-silico* analysis of AMR in *A. baumannii* genomes deposited at the NCBI

In parallel, the frequency and percentage of resistance genes were investigated in 104 whole-genome of *A. baumannii* strains of German origin deposited at the NCBI. The numbers of resistance genes conferring a specific antibiotic resistance were identified. Additionally, beta-lactamase genes were indicated and divided into their molecular group (class A, B, C, D; based on Ambler), and the plot is separated into chromosomal and plasmid DNA contigs. The identified ß-lactamases and non-ß-lactamases AMR genes in genomes, some are chromosomal-encoded, and some are plasmid-encoded genes (Fig. S1).

In total, 101 AMR genes were identified in 104 genomes. AMR genes confer resistance to cephalosporin antibiotics were the most frequent genes identified and represented by 37 different gene variants. Among them, 31 *bla*_ADC_ variants were identified, and *bla*_ADC-73_ was the most frequent gene and was found in 19 (18%) isolates, followed by *bla*ADC-30 in 15 (14.4%), and *bla*_ADC-166_ in eight (7.7%) isolates, while *bla*_ADC-25_ was seen only in two isolates (1.9%). Besides, the acquired *bla*_TEM-12_ was found in 19 (18%) isolates.

Twenty-four AMR genes conferring resistance to carbapenem compounds were identified. Among them, 19 genes belong to the intrinsic *bla*_OXA-51-like_ carbapenemase gene; of them*, bla*_OXA-66_ was the most frequent and was found in 40 (38.5%) isolates. Additionally, the *bla*_OXA-23_ was the most frequent gene found in 43 (41%) isolates, while the *bla*_NDM-1_ was found in three (2.9%) isolates. Sixteen AMR genes conferring resistance to aminoglycosides were identified. Aminoglycoside nucleotidyltransferase *ant* (3″)-IIa conferred resistance to streptomycin and spectinomycin and was found in 104 (100%) genomes. Aminoglycoside O-phosphotransferase *aph* (3″)-Ib and *aph* (6)-Id confers resistance to streptomycin were found in 48 (46%), and 45 (43%) of genomes, respectively, followed by *aph* (3′)-Ia and *aph* (3′)-VIa that were found in 28 (27%) and 24 (23%) of genomes, respectively (Table 3).

**Table 3:** Antimicrobial resistance genes detected in 104 whole-genome sequences of *A. baumannii* originating from Germany and deposited in NCBI

No.	Name of gene	Group	Frequency (*n* = 104)	Percentage 100%	Predicted Phenotype	Accession No.
1	*aph*(3′)-VI	PHT	7	6.7	Amikacin, Kanamycin	NG_051730.1
2	*aph*(3′)-VIa	PHT	24	23		NG_047448.1
3	*aph*(3′)-VIb	PHT	2	1.9		NG_047449.1
4	*aac*(6′)-Ib	ACT	3	2.9	Aminoglycoside	NG_051695.1
5	*aac*A16	ACT	6	5.7		NG_052380.1
6	*aac*(3)-I	ACT	22	21	Gentamicin	NG_047234.1
7	*aac*(3)-IId	ACT	1	0.96		NG_047251.1
8	*arm*A	MET	22	21		NG_052432.1
9	*ant*(2″)-Ia	NUT	13	12.5	Gentamicin; Kanamycin; Tobramycin	NG_047431.1
10	*aph*(3′)-Ia	PHT	28	27	Kanamycin	NG_052432.1
11	*aad*A1	NUT	22	21	Streptomycin	NG_047327.1
12	*aad*A2	NUT	1	0.96		NG_051846.1
13	*aad*A5	NUT	1	0.96		NG_047357.1
14	*aph*(3″)-Ib	PHT	48	46		NG_047413.1
15	*aph*(6)-Id	PHT	45	43		NG_047464.1
16	*ant*(3″)-IIa	PHT	104	100	Streptomycin; Spectinomycin	NG_054646.1
17	*bla*CARB-16	CARB-5	1	0.96	Beta-Lactam	NG_048718.1
18	*bla*Nmca	Class A	1	0.96		NG_055474.1
19	*bla*OXA-699	Single	1	0.96		NG_062321.1
20	*bla*OXA-735	Single	1	0.96		NG_062267.1
21	*bla*TEM-1	TEM	1	0.96		NG_050145.1
22	*ble*-MBL	BRP	3	2.9	Bleomycin	NG_047559.1
23	*bla*NDM-1	NDM	3	2.9	Carbapenem	NG_049326.1
24	*bla*OXA-100	OXA-51	6	5.7		NG_049394.1
25	*bla*OXA-104		1	0.96		NG_049397.1
26	*bla*OXA-126		1	0.96		NG_049425.1
27	*bla*OXA-208		4	3.8		NG_049506.1
28	*bla*OXA-314		1	0.96		NG_049608.1
29	*bla*OXA-317		1	0.96		NG_049611.1
30	*bla*OXA-365		1	0.96		NG_049658.1
31	*bla*OXA-374		2	1.9		NG_049665.1
32	*bla*OXA-378		2	1.9		NG_049669.1
33	*bla*OXA-430		3	2.9		NG_049717.1
34	*bla*OXA-51		1	0.96		NG_049788.1
35	*bla*OXA-64		12	11.5		NG_049804.1
36	*bla*OXA-66		40	38.5		NG_049806.1
37	*bla*OXA-68		5	4.8		NG_049808.1
38	*bla*OXA-69		11	10.6		NG_049809.1
39	*bla*OXA-88		1	0.96		NG_049828.1
40	*bla*OXA-90		2	1.9		NG_049831.1
41	*bla*OXA-94		3	2.9		NG_049835.1
42	*bla*OXA-98		1	0.96		NG_049839.1
43	*bla*OXA-23	OXA-23	43	41		NG_049525.1
44	*bla*OXA-164	OXA-58	3	2.9		NG_049463.1
45	*bla*OXA-72	OXA-40	10	9.6		NG_049813.1
46	*bla*OXA-558	Single	4	3.8		NG_054702.1
47	*cat*A1	*cat*A	9	8.6	Chloramphenicol	NG_047582.1
48	*cat*B8	*cat*B3	2	1.9		NG_047616.1
49	*cml*A1	*cml*A1	1	0.96		NG_047647.1
50	*cml*A5	*cml*A1	1	0.96		NG_051436.1
51	*cml*B1	*cml*B1	6	5.8		NG_047658.1
52	*flo*R	type E-3	3	2.8	Chloramphenicol; Florfenicol	NG_047869.1
53	*bla*ADC-101	ADC	2	1.9	Cephalosporin	NG_051440.1
54	*bla*ADC-11		3	2.9		NG_048635.1
55	*bla*ADC-117		1	0.96		NG_064676.1
56	*bla*ADC-120		3	2.9		NG_064678.1
57	*bla*ADC-154		1	0.96		NG_054996.1
58	*bla*ADC-155		2	1.9		NG_055285.1
59	*bla*ADC-156		1	0.96		NG_055286.1
60	*bla*ADC-158		1	0.96		NG_055786.1
61	*bla*ADC-160		1	0.96		NG_055788.1
62	*bla*ADC-163		1	0.96		NG_056105.1
63	*bla*ADC-165		1	0.96		NG_056107.1
64	*bla*ADC-166		8	7.7		NG_056108.1
65	*bla*ADC-167		1	0.96		NG_056109.1
66	*bla*ADC-179		1	0.96		NG_061395.1
67	*bla*ADC-184		1	0.96		NG_064707.1
68	*bla*ADC-185		1	0.96		NG_064708.1
69	*bla*ADC-186		3	2.9		NG_064709.1
70	*bla*ADC-192		1	0.96		NG_064715.1
71	*bla*ADC-25		2	1.9		NG_048649.1
72	*bla*ADC-26		6	5.8		NG_048650.1
73	*bla*ADC-30		15	14.4		NG_048652.1
74	*bla*ADC-32		2	1.9		NG_050717.1
75	*bla*ADC-57		4	3.8		NG_051494.1
76	*bla*ADC-6		1	0.96		NG_048669.1
77	*bla*ADC-73		19	18		NG_048678.1
78	*bla*ADC-74		4	3.8		NG_048679.1
79	*bla*ADC-76		5	4.8		NG_048681.1
80	*bla*ADC-79		7	6.7		NG_048684.1
81	*bla*ADC-80		1	0.96		NG_048686.1
82	*bla*ADC-95		1	0.96		NG_051459.1
83	*bla*ADC-96		1	0.96		NG_051460.1
84	*bla*CMY-30	CMY	1	0.96		NG_048825.1
85	*bla*CTX-M^−15^	CTX-M^−15^	1	0.96		NG_048935.1
86	*bla*GES-11	GES	1	0.96		NG_049113.1
87	*bla*PER-1	PER	2	1.9		NG_049960.1
88	*bla*PER-10		1	0.95		NG_059319.1
89	*bla*TEM-12	TEM	19	18		NG_050163.1
90	*mph*(E)	*mph*(E)	34	32.7	Macrolide	NG_064660.1
91	*msr*(E)	*msr*(E)	33	31.7		NG_048007.1
92	*arr*-2		1	0.96	Rifamycin	NG_048580.1
93	*sat*2_gen		4	3.8	Streptothricin	NG_048068.1
94	*sul*1		22	21	Sulfonamide	NG_048082.1
95	*sul*2		47	45		NG_051852.1
96	*Tet*.39	*tet* efflux	7	6.7	Tetracycline	NG_048137.1
97	*Tet*.A		6	5.8		NG_048154.1
98	*Tet*.B		38	36.5		NG_048163.1
99	*dfr*A1	*dfr*A	4	3.8	Trimethoprim	NG_047676.1
100	*dfr*A17		1	0.96		NG_047710.1
101	*dfr*A7		1	0.96		NG_047737.1

*BRP* bleomycin resistant protein, *ACT* acetyltransferase, *MET* methyltransferase, *NUTN* ucleotidyltransferase, *PHT* phosphotransferase

Six AMR genes confer resistance to chloramphenicol antibiotics were found. The *cat*A1 and *cml*B1 were the most frequent and found in nine (8.6%) and six (5.8%) genomes, respectively. Three AMR genes confer resistance to tetracycline compounds were identified. The *tet*. B gene was the most frequent and found in 38 (36.5%) isolates, followed by *tet*.39 and *tet*. A. Two AMR genes confer resistance to sulfonamides were identified; the *sul*1 and *sul*2 were found in 22 (21%) and 47 (45%) of genomes, respectively. Three genes encoded Trimethoprim resistance were found, and the *dfr*A1 was the most frequent and found in four (3.8%) isolates, followed by the *dfr*A7 and *dfr*A17 genes. Macrolide resistance was predominantly encoded by the *mph.* E gene in 34 (32.7%) isolates and *msr.* E in 33 (31.7%) genomes. Rifampicin resistance was encoded by *arr*-2 and was found in one strain (Table 3).

### The frequency and profiling of AMR in genomes of *A. baumannii* from Germany

As shown in Table 4, the comprehensive analysis of AMR in 189 genomes of *A. baumannii* of German origin revealed 15 AMR genes with a frequency of more than 10%. The *ant* (3″)-IIa confers resistance to aminoglycosides was the most prevalent gene with a frequency of 55%, followed by the *bla*_ADC.25_ confer resistance to cephalosporin with a frequency of 38.6%, and the two genes confer resistance to carbapenems, *bla*_OXA-23_ and *bla*_OXA-51-like_ (*bla*_OXA-66 variant),_ with a frequency of 29 and 26.5%, respectively. Around a quarter of genomes (26%) harbored *sul*2 that confer resistance to sulfonamides, while *sul*1 was found in 13.2% of the genomes. The frequency of *tet.* B gene confer resistance to tetracycline was 19.5%, and the frequency of *mph*. E and *msr.* E confer resistance to macrolide was 19%. The variants of acquired *bla*_TEM_ were found in 23 genomes with a frequency of 12% (Table 4).

**Table 4:** The total frequency and percentages of the AMR genes in 189 genomes of *A. baumannii* isolated from Germany

	AMR gene	Mechanism	Frequency ^a^ (NCBI+WGS)	Total (189)	%	Predicted phenotype
1	*ant*(3″)-IIa	Antibiotic inactivation	104 + 0	104	55%	Aminoglycosides
2	*bla*ADC.25	Ambler class C beta-lactamase	2 + 71	73	38.6%	Cephalosporins
3	*bla*OXA-23	Ambler class D beta-lactamase	43 + 12	55	29%	Carbapenems
4	*bla*OXA-66 (*bla*OXA-51-like)	Ambler class D beta-lactamase	40 + 10	50	26.5%	Carbapenems
5	*sul*2	Antibiotic target replacement	47 + 2	49	26%	Sulfonamides
6	*aph*(3″)-Ib	Antibiotic inactivation	48 + 0	48	25.3%	Aminoglycosides
7	*aph*(6)-Id	Antibiotic inactivation	45 + 9	44	23.3%	Aminoglycosides
8	*tet.* B	Antibiotic efflux	38 + 9	37	19.5%	Tetracycline
9	*mph*(E)	Enzymes inactivation	34 + 2	36	19%	Macrolide
10	*msr*(E)	Antibiotic target protection	33 + 3	36	19%	Macrolide
11	*aph*(3″)-Ia	Antibiotic inactivation	28 + 0	34	18%	Aminoglycosides
12	*sul*1	Antibiotic target replacement	22 + 3	25	13.2%	Sulfonamides
13	*aph*(3′)-VIa	Antibiotic inactivation	24 + 0	24	12.6%	Aminoglycosides
14	*bla*TEM	Antibiotic inactivation	20 + 3	23	12%	Cephalosporins
15	*bla*ADC-73	Ambler class C beta-lactamase	19 + 0	19	10%	Cephalosporins

^a^frequency of genes in genomes deposited in the NCBI (104) and 85 WGS data at our laboratory

## Discussion

The ability of *A. baumannii* to survive in adverse environmental conditions and to develop or acquire resistance make it one of the most critical nosocomial pathogens in the hospital’s environment [21]. The presence of various plasmids in the genome of *A. baumannii* [18] and its ability to acquire foreign DNA [19, 20] enhance the acquisition of AMR genes. Several reports suggested that integrons play significant roles in the horizontal transfer of AMR genes in *A. baumannii,* particularly genes that confer resistance to aminoglycosides, chloramphenicol and tetracycline [22–24]. Identification of acquired AMR genes circulating in *A. baumannii* is essential for understanding the underlying mechanisms of the acquisition and development of antimicrobial resistance. Next-generation sequence (NGS) technology became available in most routine diagnostic laboratories worldwide, and it is anticipated to substitute the traditional PCR tools for identifying AMR genes. Thus, the current study is focusing on the detection of acquired AMR genes and antimicrobial resistance profiles of 85 *A. baumannii* strains that were isolated from humans and dried milk samples in Germany and extraction of the relevant information from another 104 genomes of *A. baumannii* submitted to the NCBI from different laboratories across Germany.

Antimicrobial resistance is on the rise in foods and environmental sources. MDR *Acinetobacter* strains have been isolated from dried milk in Germany [7], infant milk formulas in Brazil [25] and China [26], as well as from bulk tank milk (BTM) samples and mastitic milk samples of dairy cattle in different districts of Korea [27, 28], representing a significant risk of the transmission of this pathogen to consumers. Inside animal hosts and in the environment, *A. baumannii* cohabits with several bacterial species. The potential acquisition of horizontal resistance genes from other bacterial species is very high due to the presence of plasmids [18]. In total, 15 AMR genes were identified in strains obtained from powdered milk samples. All milk powder samples were obtained from the end product at the production level. Thus, the origin of *A. baumannii* in milk samples is unknown because the microbes can enter the dairy supply chain at different stages during milk collection, production and processing [29]. Contamination of dried milk with *A. baumannii* and the existence of such genes is evidence of a potential threat that should be considered and can affecting human consumers. This highlights the urgent need for strict hygiene measures during the processing of dried milk.

The high frequency of resistance for carbapenems and cephalosporins was found in both groups of *A. baumannii*, either sequenced isolates or genomes deposited at NCBI. MDR strains harboring diverse resistance genes confer resistance for carbapenems and cephalosporins were isolated in various hospital outbreaks in Germany [30–32]. Broad diversity of OXA-type carbapenemase genes was identified, and the *bla*_OXA-23_ and *bla*_OXA-51-like_ (*bla*_OXA-66 variant_) were among the most frequent. Both are ambler class D ß-lactamases, which originally relatively rare and always plasmid-mediated. It is worth mentioning that the OXA β-lactamase group was among the earliest β-lactamases detected, and the variants OXA-23 and OXA-51 are currently spreading on plasmids. Therefore their transmission between different bacterial species can be reasonably assumed [33]. Several studies have shown that the presence of one or both of those genes in *A. baumannii* is associated with resistance to all β-lactam antibiotics, including carbapenems [34–37]. The class D carbapenemase *bla*_OXA-66/OXA-51-like_ contributes to intrinsic resistance to imipenem in clinical strains of *A. baumannii* [38]. The *bla*_OXA-51_ was detected initially in *A. baumannii* from Argentina in 1996 [39]. It is the largest group of intrinsic OXA-type β-lactamases identified and became an important marker for species identification of *A. baumannii.* Association of IS*Aba*1 with *bla*_OXA-51-like_ can increase its expression levels by 50-fold [33]. The oxacillinase *bla*_OXA-23_ was identified for the first time in *A. baumannii* strains isolated from the United Kingdom in 1993. Later, it has been found and linked to the dissemination of carbapenem-resistant in *A. baumannii* worldwide [40] and is one of the most dominant resistance genes described in *A. baumannii* in Germany last decade [5].

The ADC beta-lactamases are cephalosporinase with extended-spectrum resistance to cephalosporins. Thirty-one ADC beta-lactamases variants were found in isolates deposited at NCBI, and the *bla*_ADC-73_ was the most frequent. The *bla*_ADC-73_ is a novel variant of *bla*_ADC_ and has been detected in *A. baumannii* isolates in a few studies [41, 42]. Proteogenomic analysis of XDR strains showed that *bla*_ADC-73_ is one of the significant determinants responsible for antibiotic resistance in *A. baumannii* [43]. The presence of the IS*Aba*1 element in *bla*_ADC–73_ gene is responsible for increase the cephalosporinase gene expression [44]. In contrast, *bla*_ADC-25_1_ was the only variant identified in the 85 sequenced isolates and was found in all isolates (100%). The cephalosporinase-encoding *bla*_ADC-25-like_ gene was uncommon in Germany; however, it has been detected in hospital-acquired *A. baumannii* infection [31]. It is worth mentioning that the *ant* (3″)-IIa conferring resistance to aminoglycosides was found in all isolates (*n* = 104) deposited in the NCBI database. However, none of the 85 sequenced isolates contained this gene by using the ResFinder server. The comprehensive ResFinder server was used for the detection of acquired resistance genes in the sequenced isolates and failed to detect the *ant* (3″)-IIa. Searching for non-β-lactamases intrinsic resistance genes using CARD and NCBI databases succeeded in detecting this gene in all sequenced isolates [11]. Thus, this study highlights the necessity of combining different databases to determine the resistance profiles of *A. baumannii* isolates and depending on one database to discriminate the presence of all AMR genes was insufficient [11].

Three tetracycline-encoding genes were identified in *A. baumannii*, and *tet.* B was the most frequent in both groups. The *tet.* B is a tetracycline efflux protein expressed in various Gram-negative bacteria. It is a major facilitator superfamily (MFS) antibiotic efflux pump that confers tetracycline resistance but not tigecycline [45]. In our survey, it was found in nine sequenced isolates; among them, only eight were tigecycline resistant. Tigecycline is a glycylcycline developed to help overcome tetracycline-resistant in microorganisms [46]. In *A. baumannii,* it was reported that *tet.* A plays an essential role in tigecycline efflux by removing and transporting tigecycline from the cytoplasm to the periplasm [47]. The *tet*. A.6 was identified in a tigecycline resistant strain of human origin and was present in 5.8% of genomes deposited in NCBI. Two genes, the *sul*1 and *sul*2 mediated resistance to sulfonamides were identified. Both are mediated by transposons and plasmids and are express dihydropteroate synthases in Gram-negative bacteria that confer resistance to sulfonamides [48]. The presence of one or both genes in *A. baumannii* isolates conferred resistance to trimethoprim/sulfamethoxazole.

In spite, all sequenced *A. baumannii* isolates (100%) in the current study were chloramphenicol resistant; only four isolates harbored chloramphenicol acetyltransferase encoded variant of the *cat* genes and chloramphenicol exporter *flo*R gene. It was indicated previously that most *A. baumannii* isolates are intrinsically resistant to chloramphenicol; however, the mechanism responsible for such resistance is not apparent yet [49]. Three isolates were colistin-resistant; however, none of the plasmid-mediated resistance to colistin (*mcr* genes) was identified. The mechanism of resistance to colistin in *A. baumannii* is associated with the mutation in the protein *Pmr*AB [50].

## Conclusion

*Acinetobacter baumannii* is an important opportunistic nosocomial pathogen in healthcare settings in Germany. AMR genes were investigated in the genome of 189 German *A. baumannii* strains. The spread of MGE is the main driving force in the spread and dissemination of acquired resistance, but a chromosomal gene mutation is a possible route. Three major known resistance mechanisms are associated with MGE, i.e., enzyme inactivation, antibiotics efflux, and antibiotic target sites’ replacement. Acquired AMR belonging to those mechanisms was seen in the current studied group of *A. baumannii.* Understanding the genetic mobilization of AMR genes in *A. baumannii* collected from different reservoirs is essential to investigate resistance genes’ interspecies mobility. This is paramount in preventing dissemination and spillover. The presence of *A. baumannii* strains harboring divers acquired AMR genes in milk powder raises safety and health concerns and highlights the need for a more hygienic environment for the processing of dried milk.

## Materials and methods

### Molecular characterization and phenotyping of *A. baumannii* strains

Eighty-five *A. baumannii* strains isolated between 2005 and 2018 in Germany were received by the Institute of Bacterial Infections and Zoonoses (IBIZ, Jena) for confirmation and typing. Fourteen clinical strains were isolated from humans between 2017 and 2018, and 71 non-clinical strains were obtained from powdered milk samples produced in Germany. All milk powder samples investigated in the current study were isolated from the end product of three different companies in Germany between 2005 and 2012 at the production level. The strains were identified at species level using a combination of Matrix-Assisted Laser Desorption/Ionization Mass Spectrometry (MALDI-TOF MS) with a score value > 2.300 and the intrinsic *bla*_OXA-51-like_-PCR [51]. The identity and non-clonality of all isolates were confirmed using the WGS data. Antimicrobial susceptibility testing (AST) for 18 antibiotics was carried out via the broth microdilution method using an automated MICRONAUT-S system (Micronaut, MERLIN Diagnostics GmbH, Bornheim-Hersel Germany) according to the manufacturer’s instructions. The minimum inhibitory concentration (MIC) was determined according to the Clinical and Laboratory Standards Institute (CLSI) breakpoint guidelines available for *A. baumannii*, as previously described [11].

### WGS based detection of acquired AMR genes in *A. baumannii* strains

DNA was extracted using the High Pure PCR Template Preparation Kit (Roche Diagnostics GmbH, Mannheim, Germany) according to the manufacturer’s instructions. The sequencing library was prepared, followed by paired-end sequencing on an Illumina MiSeq sequencer (Illumina, USA). The raw sequencing data were assembled and analyzed as previously described [11]. The comprehensive ResFinder server [52] was used to identify the acquired AMR genes among *A. baumannii* strains. Known acquired resistance genes relevant to ß-lactams (including carbapenems and cephalosporins), aminoglycosides, phenicoles, macrolide-lincosamide-streptogramin B, quinolones, sulfonamides, and tetracyclines were included in the analysis. The β-lactamase and non-β-lactamase gene variants were determined with a 100% identity using the *A. baumannii* reference genome (Accession ASM74664v1) as input. Reference sequences for acquired resistance genes were curated from those described in the ResFinder (https://cge.cbs.dtu.dk/services/data.php) datasets.

### WGS based detection of AMR genes in *A. baumannii* genomes

In parallel, 104 out of 9.579 available genomes of *A. baumannii* were downloaded from the NCBI database https://www.ncbi.nlm.nih.gov/genome/browse/#!/ prokaryotes/403/ (access date 10.09.2020). *Acinetobacter baumannii* genomes with the Genbank tag (/country=), which contained “Germany” were eligible for inclusion. In this way, we extracted 104 out of 195 German *A. baumannii*. The extracted isolates were mostly clinical isolates from 2012 to 2019, and in 88 out of 104 isolates, an isolation source was specified as the following: 19 groins, 12 wounds, 10 wound swab, 9 rectal swabs, 7 tracheal secretions, 4 respiratory, 3 blood, 3 clinical material, 2 bronchial secretions, 2 screening swab, 1 catheter swab, 1 catheter urine, 1 cerebrospinal fluid, 1 conjunctivitis, 1 drainage liquid, 1 groin swab, 1 perianal swab, 1 pleural drainage, 1 respiratory tract, 1 sterile tissue, 1 stoma swab, 1 throat, 1 tracheal secretion, 1 urine, 2 water and 1 eggshell. These sequences were annotated with ABRicate v.1.0.1 (https://github.com/tseemann/abricate). The NCBI AMR Finder Plus [53], the ResFinder database [52], the CARD database [54] and the ARG-ANNOT [55] were used for the identification of resistance genes. Only resistance genes with a coverage of > 80 and > 75% identity (proportion of exact nucleotide matches) were accepted. The following information was extracted from the data: the gene’s names, frequency within 104 genomes, percentage, predicted phenotype and accession number for each gene. DNA contigs were separated via plasflow (v1.1.0) into chromosomal and plasmid contigs. Gene detection was performed via abricate and fargene (https://microbiomejournal.biomedcentral.com/articles/10.1186/s40168-019-0670-1), and plotted via ggplot2.

## Supplementary Information

**Additional file 1: Figure S1**. *A. baumannii* genomes are listed at the x-axis. Resistance against antibiotics is indicated on the y-axis. The circle sizes and colours represent the number of resistance genes identified, conferring a specific antibiotic resistance. Additionally, beta-lactamase genes are also indicated and are divided into their molecular group (class A, B, C, D; based on Ambler) due to their importance. The plot is separated into chromosomal and plasmid DNA contigs.

**Additional file 2: Supplementary Table 1.** List of sequenced 85 A. baumannii strains showing ID, year of isolation and source of each strain, and the full details of acquired resistance genes that have been identified by ResFinder server.

## Data Availability

All data generated or analyzed during this study are included in this published article and its supplementary information files.

## Abbreviations

AMR: Antimicrobial resistance
MDR: Multidrug-resistant
XDR: Extensively drug-resistant
PDR: Pan-drug resistant
AST: Antimicrobial susceptibility testing
MGEs: Mobile genetic elements
NGS: Next-generation sequencing
WGS: Whole-genome sequencing
MIC: Minimal inhibitory concentration
*A. baumannii*: *Acinetobacter baumannii*
CRAB: Carbapenem-resistant *Acinetobacter baumannii*
TEM: Temoneira
SHV: Sulfhydryl variable
CTX-M: Cefotaxime hydrolyzing capabilities
OXA: Oxacillinase

## References

[1] Santajit S, Indrawattana N (2016). Mechanisms of antimicrobial resistance in ESKAPE pathogens. Biomed Res Int.

[2] Ayobami O, Willrich N, Harder T, Okeke IN, Eckmanns T, Markwart R (2019). The incidence and prevalence of hospital-acquired (carbapenem-resistant) *Acinetobacter baumannii* in Europe, eastern Mediterranean and Africa: a systematic review and meta-analysis. Emerg Microbes Infect.

[3] Sleiman A, Fayad AGA, Banna H, Matar GM. Prevalence and molecular epidemiology of carbapenem-resistant Gram-negative Bacilli and their resistance determinants in the Eastern Mediterranean region over the last decade. J Glob Antimicrob Resist. 2021;25:209–21. 10.1016/j.jgar.2021.02.033. 10.1016/j.jgar.2021.02.033 33812049

[4] Babu Rajendran N, Mutters NT, Marasca G, Conti M, Sifakis F, Vuong C (2020). Mandatory surveillance and outbreaks reporting of the WHO priority pathogens for research & discovery of new antibiotics in European countries. Clin Microbiol Infect.

[5] Wareth G, Brandt C, Sprague LD, Neubauer H, Pletz MW. Spatio-temporal distribution of *Acinetobacter baumannii* in Germany-a comprehensive systematic review of studies on resistance development in humans (2000–2018). Microorganisms. 2020;8(3):375. 10.3390/microorganisms8030375. 10.3390/microorganisms8030375 PMC7143851 32155886

[6] Ewers C, Klotz P, Leidner U, Stamm I, Prenger-Berninghoff E, Göttig S, Semmler T, Scheufen S (2017). OXA-23 and ISAba1-OXA-66 class D β-lactamases in *Acinetobacter baumannii* isolates from companion animals. Int J Antimicrob Agents.

[7] Cho GS, Li B, Rostalsky A, Fiedler G, Rösch N, Igbinosa E, Kabisch J, Bockelmann W, Hammer P, Huys G, Franz CMAP (2018). Diversity and antibiotic susceptibility of *Acinetobacter* strains from milk powder produced in Germany. Front Microbiol.

[8] Pulami D, Schauss T, Eisenberg T, Wilharm G, Blom J, Goesmann A, et al. *Acinetobacter baumannii* in manure and anaerobic digestates of German biogas plants. FEMS Microbiol Ecol. 2020;96(10):fiaa176. 10.1093/femsec/fiaa176. 10.1093/femsec/fiaa176 32832994

[9] Alexander J, Hembach N, Schwartz T (2020). Evaluation of antibiotic resistance dissemination by wastewater treatment plant effluents with different catchment areas in Germany. Sci Rep.

[10] Wareth G, Neubauer H, Sprague LD (2019). *Acinetobacter baumannii* - a neglected pathogen in veterinary and environmental health in Germany. Vet Res Commun.

[11] Wareth G, Linde J, Hammer P, Nguyen NH, Nguyen TNM, Splettstoesser WD, et al. Phenotypic and WGS-derived antimicrobial resistance profiles of clinical and non-clinical *Acinetobacter baumannii* isolates from Germany and Vietnam. Int J Antimicrob Agents. 2020;56(4):106127. 10.1016/j.ijantimicag.2020.106127. 10.1016/j.ijantimicag.2020.106127 32750418

[12] Brovedan MA, Cameranesi MM, Limansky AS, Morán-Barrio J, Marchiaro P, Repizo GD (2020). What do we know about plasmids carried by members of the *Acinetobacter* genus?. World J Microbiol Biotechnol.

[13] Pagano M, Martins AF, Barth AL (2016). Mobile genetic elements related to carbapenem resistance in *Acinetobacter baumannii*. Brazilian J Microbiol.

[14] Brandt C, Braun SD, Stein C, Slickers P, Ehricht R, Pletz MW, Makarewicz O (2017). In silico serine β-lactamases analysis reveals a huge potential resistome in environmental and pathogenic species. Sci Rep.

[15] Chakravarty B (2020). Genetic mechanisms of antibiotic resistance and virulence in *Acinetobacter baumannii*: background, challenges and future prospects. Mol Biol Rep.

[16] Blackwell GA, Hamidian M, Hall RM. IncM plasmid R1215 is the source of chromosomally located regions containing multiple antibiotic resistance genes in the globally disseminated *Acinetobacter baumannii* GC1 and GC2 clones. mSphere. 2016;1(3):e00117–16. 10.1128/mSphere.00117-16. 10.1128/mSphere.00117-16 PMC4899885 27303751

[17] Wong D, Nielsen TB, Bonomo RA, Pantapalangkoor P, Luna B, Spellberg B (2017). Clinical and pathophysiological overview of *Acinetobacter* infections: a century of challenges. Clin Microbiol Rev.

[18] Salgado-Camargo AD, Castro-Jaimes S, Gutierrez-Rios RM, Lozano LF, Altamirano-Pacheco L, Silva-Sanchez J, Pérez-Oseguera Á, Volkow P, Castillo-Ramírez S, Cevallos MA (2020). Structure and evolution of *Acinetobacter baumannii* plasmids. Front Microbiol.

[19] Krizova L, Dijkshoorn L, Nemec A (2011). Diversity and evolution of AbaR genomic resistance islands in *Acinetobacter baumannii* strains of European clone I. Antimicrob Agents Chemother.

[20] Lin MF, Lan CY (2014). Antimicrobial resistance in *Acinetobacter baumannii:* from bench to bedside. World J Clin Cases.

[21] Nowak P, Paluchowska P (2016). *Acinetobacter baumannii*: biology and drug resistance - role of carbapenemases. Folia Histochem Cytobiol.

[22] Lin MF, Chang KC, Yang CY, Yang CM, Xiao CC, Kuo HY, Liou ML (2010). Role of integrons in antimicrobial susceptibility patterns of *Acinetobacter baumannii*. Jpn J Infect Dis.

[23] Huang LY, Chen TL, Lu PL, Tsai CA, Cho WL, Chang FY, Fung CP, Siu LK (2008). Dissemination of multidrug-resistant, class 1 integron-carrying *Acinetobacter baumannii* isolates in Taiwan. Clin Microbiol Infect.

[24] Rabea RA, Zaki MES, Fahmy EM, Fathelbab A. Molecular study of nodulation division genes and integron genes in *Acinetobacter baumannii.* Clin Lab. 2020;66(9). 10.7754/Clin.Lab.2020.200124. 10.7754/Clin.Lab.2020.200124 32902213

[25] Araújo BC, Moraes MS, Costa LE, Nascimento JS (2015). Short communication: multidrug-resistant *Acinetobacter baumannii*-*calcoaceticus* complex isolated from infant milk formula and utensils in a nursery in Rio de Janeiro, Brazil. J Dairy Sci.

[26] Wu S, Jiang Y, Lou B, Feng J, Zhou Y, Guo L, Forsythe SJ, Man C (2018). Microbial community structure and distribution in the air of a powdered infant formula factory based on cultivation and high-throughput sequence methods. J Dairy Sci.

[27] Gurung M, Nam HM, Tamang MD, Chae MH, Jang GC, Jung SC, Lim SK (2013). Prevalence and antimicrobial susceptibility of *Acinetobacter* from raw bulk tank milk in Korea. J Dairy Sci.

[28] Nam HM, Lim SK, Kim JM, Joo YS, Jang KC, Jung SC (2010). In vitro activities of antimicrobials against six important species of gram-negative bacteria isolated from raw milk samples in Korea. Foodborne Pathog Dis.

[29] McHugh AJ, Feehily C, Fenelon MA, Gleeson D, Hill C, Cotter PD. Tracking the dairy microbiota from farm bulk tank to skimmed milk powder. mSystems. 2020;5(2):e00226–20. 10.1128/mSystems.00226-20. 10.1128/mSystems.00226-20 PMC7141888 32265313

[30] Katchanov J, Asar L, Klupp EM, Both A, Rothe C, König C, Rohde H, Kluge S, Maurer FP (2018). Carbapenem-resistant gram-negative pathogens in a German university medical center: prevalence, clinical implications and the role of novel β-lactam/β-lactamase inhibitor combinations. Plos One.

[31] Wendel AF, Malecki M, Otchwemah R, Tellez-Castillo CJ, Sakka SG, Mattner F (2018). One-year molecular surveillance of carbapenem-susceptible *A. baumannii* on a German intensive care unit: diversity or clonality. Antimicrob Resist Infect Control.

[32] Rieber H, Frontzek A, Pfeifer Y (2017). Molecular Investigation of carbapenem-resistant *Acinetobacter* spp. from hospitals in North Rhine-Westphalia, Germany. Microbial Drug Resist (Larchmont).

[33] Evans BA, Amyes SG (2014). OXA β-lactamases. Clin Microbiol Rev.

[34] Khurshid M, Rasool MH, Ashfaq UA, Aslam B, Waseem M, Xu Q, Zhang X, Guo Q, Wang M (2020). Dissemination of Bla (OXA-23)-harbouring carbapenem-resistant *Acinetobacter baumannii* clones in Pakistan. J Glob Antimicrob Resist.

[35] Hu S, Niu L, Zhao F, Yan L, Nong J, Wang C, Gao N, Zhu X, Wu L, Bo T, Wang H, Gu J (2019). Identification of *Acinetobacter baumannii* and its carbapenem-resistant gene Bla (OXA-23-like) by multiple cross displacement amplification combined with lateral flow biosensor. Sci Rep.

[36] Rao M, Rashid FA, Shukor S, Hashim R, Ahmad N. Detection of antimicrobial resistance genes associated with carbapenem resistance from the whole-genome sequence of *Acinetobacter baumannii* isolates from Malaysia. Can J Infect Dis Med Microbiol. 2020;2020:5021064. 10.1155/2020/5021064. 10.1155/2020/5021064 PMC7154965 32318127

[37] Chen CM, Liu PY, Ke SC, Wu HJ, Wu LT (2009). Investigation of carbapenem-resistant *Acinetobacter baumannii* isolates in a district hospital in Taiwan. Diagn Microbiol Infect Dis.

[38] Hu WS, Yao S-M, Fung C-P, Hsieh Y-P, Liu C-P, Lin J-F (2007). An OXA-66/OXA-51-like carbapenemase and possibly an efflux pump are associated with resistance to imipenem in *Acinetobacter baumannii*. Antimicrob Agents Chemother.

[39] Brown S, Young HK, Amyes SG (2005). Characterisation of OXA-51, a novel class D carbapenemase found in genetically unrelated clinical strains of *Acinetobacter baumannii* from Argentina. Clin Microbiol Infect.

[40] Mugnier PD, Poirel L, Naas T, Nordmann P (2010). Worldwide dissemination of the *bla*OXA-23 carbapenemase gene of *Acinetobacter baumannii*. Emerg Infect Dis.

[41] Karah N, Dwibedi CK, Sjöström K, Edquist P, Johansson A, Wai SN, Uhlin BE (2016). Novel aminoglycoside resistance transposons and transposon-derived circular forms detected in carbapenem-resistant *Acinetobacter baumannii* clinical isolates. Antimicrob Agents Chemother.

[42] Hadjadj L, Shoja S, Diene SM, Rolain JM (2018). Dual infections of two carbapenemase-producing *Acinetobacter baumannii* clinical strains isolated from the same blood culture sample of a patient in Iran. Antimicrob Resist Infect Control.

[43] Lee SY, Oh MH, Yun SH, Choi CW, Park EC, Song HS, Lee H, Yi YS, Shin J, Chung C, Moon JY, Lee JC, Kim GH, Kim SI (2018). Genomic characterization of extensively drug-resistant *Acinetobacter baumannii* strain, KAB03 belonging to ST451 from Korea. Infect Genet Evol.

[44] Héritier C, Poirel L, Nordmann P (2006). Cephalosporinase over-expression resulting from insertion of ISAba1 in *Acinetobacter baumannii*. Clin Microbiol Infect.

[45] Roberts MC (2005). Update on acquired tetracycline resistance genes. FEMS Microbiol Lett.

[46] Greer ND. Tigecycline (Tygacil): The first in the glycylcycline class of antibiotics. Proc (Baylor Univ Med Cent). 2006;19(2):155–61. 10.1080/08998280.2006.11928154. 10.1080/08998280.2006.11928154 PMC1426172 16609746

[47] Foong WE, Wilhelm J, Tam HK, Pos KM (2020). Tigecycline efflux in *Acinetobacter baumannii* is mediated by TetA in synergy with RND-type efflux transporters. J Antimicrob Chemother.

[48] Sköld O (2001). Resistance to trimethoprim and sulfonamides. Vet Res.

[49] Roca I, Marti S, Espinal P, Martínez P, Gibert I, Vila J (2009). CraA, a major facilitator superfamily efflux pump associated with chloramphenicol resistance in *Acinetobacter baumannii*. Antimicrob Agents Chemother.

[50] Adams MD, Nickel GC, Bajaksouzian S, Lavender H, Murthy AR, Jacobs MR, Bonomo RA (2009). Resistance to colistin in *Acinetobacter baumannii* associated with mutations in the PmrAB two-component system. Antimicrob Agents Chemother.

[51] Turton JF, Woodford N, Glover J, Yarde S, Kaufmann ME, Pitt TL (2006). Identification of *Acinetobacter baumannii* by detection of the *bla*OXA-51-like carbapenemase gene intrinsic to this species. J Clin Microbiol.

[52] Zankari E, Hasman H, Cosentino S, Vestergaard M, Rasmussen S, Lund O, Aarestrup FM, Larsen MV (2012). Identification of acquired antimicrobial resistance genes. J Antimicrob Chemother.

[53] Feldgarden M, Brover V, Haft DH, Prasad AB, Slotta DJ, Tolstoy I, et al. Validating the AMRFinder tool and resistance gene database by using antimicrobial resistance genotype-phenotype correlations in a collection of isolates. Antimicrob Agents Chemother. 2019;63(11):e00483-19. 10.1128/AAC.00483-19. 10.1128/AAC.00483-19 PMC6811410 31427293

[54] Jia B, Raphenya AR, Alcock B, Waglechner N, Guo P, Tsang KK, Lago BA, Dave BM, Pereira S, Sharma AN, Doshi S, Courtot M, Lo R, Williams LE, Frye JG, Elsayegh T, Sardar D, Westman EL, Pawlowski AC, Johnson TA, Brinkman FSL, Wright GD, McArthur AG (2017). CARD 2017: expansion and model-centric curation of the comprehensive antibiotic resistance database. Nucleic Acids Res.

[55] Gupta SK, Padmanabhan BR, Diene SM, Lopez-Rojas R, Kempf M, Landraud L, Rolain JM (2014). ARG-ANNOT, a new bioinformatic tool to discover antibiotic resistance genes in bacterial genomes. Antimicrob Agents Chemother.

