# Supplementary figures and images for: WGS based analysis of acquired antimicrobial resistance in human and non-human Acinetobacter baumannii isolates from a German perspective
Source: BMC Microbiol. 2021 Jul 10;21:210. doi: 10.1186/s12866-021-02270-7 (PMC8272256; doi:10.1186/s12866-021-02270-7)

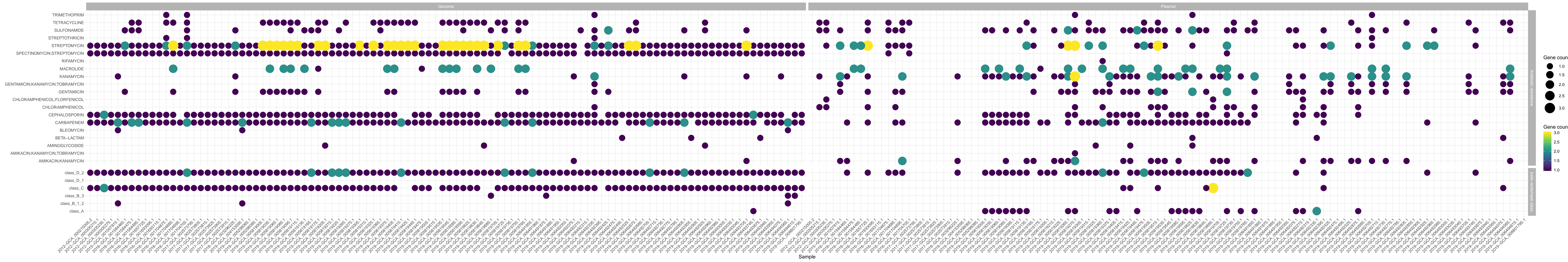

Supplement: Supplementary file 1 — Additional file 1: Figure S1. A. baumannii genomes are listed at the x-axis. Resistance against antibiotics is indicated on the y-axis. The circle sizes and colours represent the number of resistance genes identified, conferring a specific antibiotic resistance. Additionally, beta-lactamase genes are also indicated and are divided into their molecular group (class A, B, C, D; based on Ambler) due to their importance. The plot is separated into chromosomal and plasmid DNA contigs. [file 12866_2021_2270_MOESM1_ESM.pdf]
